# Rationale and design of the impact of anticoagulation therapy on the Cognitive Decline and Dementia in Patients with Nonvalvular Atrial Fibrillation (CAF) Trial: A Vanguard study

**DOI:** 10.1002/clc.23181

**Published:** 2019-04-10

**Authors:** T. Jared Bunch, Victoria Jacobs, Heidi May, Scott M. Stevens, Brian Crandall, Michael Cutler, John D. Day, Charles Mallender, Jeff Olson, Jeffrey Osborn, J. Peter Weiss, Scott C. Woller

**Affiliations:** ^1^ Intermountain Heart Institute Intermountain Medical Center Murray Utah; ^2^ Stanford University Department of Internal Medicine Palo Alto California; ^3^ Department of General Internal Medicine University of Utah School of Medicine Salt Lake City Utah

**Keywords:** atrial fibrillation, stroke prevention

## Abstract

Atrial fibrillation (AF) is associated with a risk for cognitive impairment and dementia, which is more pronounced in patients with a history of clinical stroke. Observational trials suggest that the implementation and quality of long‐term anticoagulation impact dementia risk. Emerging evidence suggests that direct oral anticoagulants may improve long‐term risk of dementia in AF patients. This manuscript describes the rational and trial design of the the Cognitive Decline and Dementia in Atrial Fibrillation Patients (CAF) Trial. CAF investigates if AF patients randomized to dabigatran etexilate will have long‐term higher cognition scores and lower rates of dementia compared in the long term to dose‐adjusted warfarin (International Normalized Ratio [INR]: 2.0‐3.0). As of 27 February 2019, a total of 120 subjects will be enrolled at one investigational site in the United States and will be followed for 2 years after study enrollment. To date, 97 have been enrolled. The average age is 74.2 years, 53% are male, and 9% had a prior stroke. In this Vanguard study, patients will be followed for 2 years after study enrollment. These prospective, randomized data will inform the understanding of two anticoagulants in AF patients as it relates to risk of cognitive decline and dementia. Cranial imaging and biomarkers collected will assist in understanding mechanisms of brain injury.

## 1 BACKGROUND AND RATIONALE

Atrial fibrillation (AF) is associated with a risk for cognitive impairment and dementia, which is more pronounced in patients with a history of clinical stroke. The relative risk of cognitive impairment and dementia among patients with AF and a prior stroke ranges from 2.43 to 2.70.^1^, ^2^ The association of AF with cognitive impairment and dementia in patients without a history of overt stroke may in part be due to subclinical cerebral infarcts, as patients with AF have a greater than 2‐fold increase in the risk of developing silent cerebral infarcts.^3^, ^4^, ^5^

Anticoagulation therapy remains the cornerstone of stroke risk reduction in most AF patients. In observational studies, warfarin anticoagulation efficacy impacts long‐term risk of dementia in AF patients without baseline dementia. Specifically, the time in therapeutic range (TTR), a marker of warfarin anticoagulation quality is significantly correlated with incident dementia in patients with AF. In a study of 2605 AF patients, decreasing categories of TTR were associated with increased dementia risk (vs >75%): <25%: hazard ratio (HR) 5.34, *P* < 0.0001; 26%‐50%: HR 4.10, *P* < 0.0001; and 51%‐75%: HR = 2.57, *P* = 0.001.^6^ The risk association was observed in patients exposed to both under‐ and over‐anticoagulation.^6^

Given the challenges in maintaining high‐quality anticoagulation among patients taking warfarin, direct oral anticoagulants (DOACs) are an attractive alternative. In patients with AF, DOACs have compared favorably to warfarin, with noninferior or superior rates of stroke and intracranial bleeding.^7^, ^8^, ^9^, ^10^ In a recent community‐based observational study of 5254 patients taking a DOAC or warfarin, the composite outcome of stroke, transient ischemic attack, or dementia was 43% more likely in the warfarin group when compared to the DOAC group. Dementia alone was more prevalent in the warfarin group, when compared to DOAC group, 0.7% vs 0.3%, *P* = 0.03, respectively.^11^ These data are observational, but are hypothesis forming in regard to the role of anticoagulation therapies use and efficacy and risk of cognitive decline in AF patients.

Dabigatran etexilate, an oral direct thrombin inhibitor, was compared to warfarin in patients with nonvalvular AF.^7^ Over a median follow‐up of 2 years, the primary endpoint of stroke or systemic embolism was reduced in the dabigatran etexilate‐treated groups (3.4% per year with warfarin compared with 3% per year with 110 mg of dabigatran etexilate and 2.2% per year with 150 mg of dabigatran etexilate). The rate of hemorrhagic stroke was reduced significantly with dabigatran etexilate compared to warfarin (HR 0.26 [95% confidence interval: 0.14‐0.49]). Major bleeding rates were also lower in the dabigatran etexilate treated groups—in particular those attributed to intracranial bleed. These favorable outcomes correlated with a mortality reduction in patients treated with dabigatran etexilate.^7^ The reduced rate of clinically overt stroke in patients on dabigatran etexilate may be accompanied by a reduction in the rate of silent ischemic events, with an accompanying reduction in incident dementia.^12^ In a community analysis of DOAC therapies, dabigatran etexilate had the lowest observed dementia rates despite the longest duration of follow‐up.^11^ No significant difference in dementia risk was reported comparing DOAC therapy with warfarin upon propensity analysis in a large Swedish registry. Indeed, the general dementia incidence rates with DOAC vs warfarin therapies per 100 years studied trend were lower with DOACs (1.13 vs 2.26). However DOAC therapy recipients were different from the overall population and the use of these newer anticoagulants was low (3% of the entire population).^13^ Although cranial injury from micro‐ and macro‐emboli appears to be a central mechanism behind the association of AF and dementia, the role of anticoagulation use and efficacy as well as the type of anticoagulation to use to prevent cognitive decline is not known.^14^

The **C**ognitive Decline and Dementia in **A**trial **F**ibrillation Patients (CAF) Trial (ClinicalTrials.gov Identifier: NCT03061006) is proposed to determine if AF patients randomized to dabigatran etexilate will have long‐term higher cognition scores and lower rates of dementia compared to dose‐adjusted warfarin (INR: 2.0‐3.0). The results of this vanguard study may inform further research design and outcome estimates.

## 2 METHODS

### 2.1 Study objectives and design

The CAF trial has two primary objectives. First, compare the rates of incident dementia and cognitive decline in patients on long‐term anticoagulation therapy with dabigatran etexilate (150 or 75 mg BID, dose based upon renal clearance) vs dose‐adjusted warfarin. Second, compare the rates of micro‐ and macro‐cerebral ischemic events in patients taking dabigatran etexilate vs those taking warfarin.

The secondary objectives will report cognitive outcomes, incident dementia, and percent of patients that develop moderate cognitive decline compared to rates observed in the general population. Also, the feasibility of recruitment, retention, compliance with study procedures, outcomes assessments, and budget will be reported. These results may inform future trial design and planning; and if feasibility goals are met, the study may serve as a vanguard for a future definitive trial.

This is a randomized, prospective, open‐label clinical study with blinded endpoint assessment. Patients with moderate‐ to high‐risk (defined as a CHADS_2_ score or CHA_2_DS_2_‐Vasc score of ≥2)^15^ nonvalvular AF will be randomly assigned to dabigatran etexilate or warfarin, adjusted to a target INR of 2.0‐3.0. The primary endpoint is incident dementia or moderate cognitive decline at 24 months. The study was filed under a physician IND (investigation new drug) application.

#### 2.1.1 Study flow diagram

**Figure 1:**
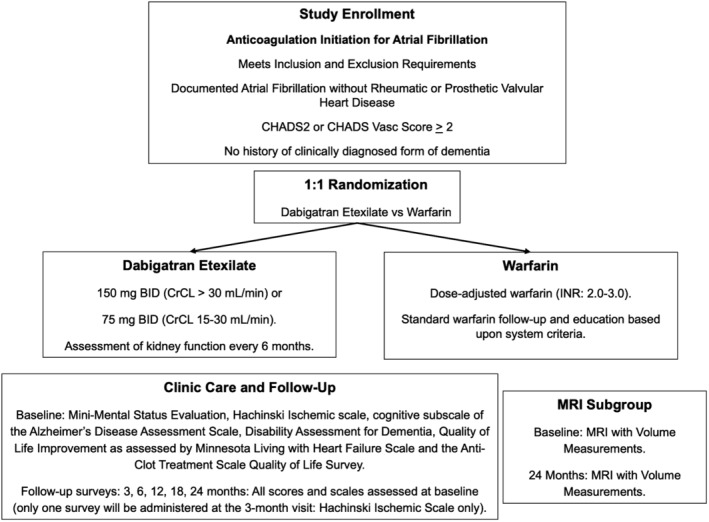
Study flow diagram illustrating enrollment, randomization, dosing, and cognitive assessment

The study flow diagram is shown in Figure 1.

#### 2.1.2 Study duration

The study will last for approximately 48 months (a target of up to 24 months to recruit the required subjects, with 24 months of follow‐up to complete the study for each subject).

#### 2.1.3 Study population

Eligible patients must meet all of the criteria listed in supplemental file, appendix A. In brief, patients will be ≥65 years of age and have AF in the absence of significant valvular heart disease as defined by the study as nonrheumatic valve disease, lack of a prosthetic valve, or lack of valve disease that may prompt valve replacement during the study duration. Patients must be able to take oral anticoagulation, complete serial testing of cognition and functional status, and have a moderate risk of thromboembolism at enrollment (CHADS_2_ score or CHA_2_DS2‐Vasc score of ≥2).

Patients will be excluded (supplemental file, appendix B) if they do not meet inclusion criteria, have severe renal dysfunction that impairs use of anticoagulation, have a history of any form of dementia that has been formally diagnosed with associated negative impact on quality of life, or have a life expectancy less than 24 months.

### 2.2 Sample size calculation and number of study subjects

Estimates of potential cognition benefit are derived from our prior work on percent TTR and dementia.^6^ In this comparative study, all patients had no history of dementia and were closely managed at the Clinical Pharmacist Anticoagulation Service (CPAS) clinics at the time of enrollment. The percent TTR averaged 62.7 ± 22.9%, with the percent of INRs being <2.0 was 25.9 ± 19.7% and the percent of INRs being >3.0 was 16.0 ± 14.6%. We have previously reported that the CPAS model using a standardized approach to chronic anticoagulation management adopting computerized clinical decision‐support has been associated with improving TTR and clinical outcomes.^16^ Because of this we anticipate the dementia outcomes will likely represent a best‐case scenario with warfarin, and may be worse in routine clinical care without warfarin dosing clinical decision support. We anticipate that dabigatran etexilate will have cognitive outcomes as good or better than the best managed warfarin patients. Among patients that had a percent TTR >75% the estimated dementia risk was 1.9%. Within our population of AF patients managed by CPAS, the distribution of patients in each category of percent TTR was: ≤25%: 215 (8.0%), 26%‐50%: 403 (15.0%), 51%‐75%: 1283 (47.6%), >75%: 792 (29.4%). Given this distribution, the estimated dementia risk of the entire population is from 4.9% to 5.5%. In those with concurrent use of an antiplatelet agent, this risk increases slightly to 5.8%. Therefore, we anticipate the dabigatran etexilate group will have a 2‐year dementia rate of 1.9% vs 5.2% in the dose adjusted warfarin population. We acknowledge that superior TTR has been associated with a suspected diminution in benefit of DOACs compared with warfarin for the outcome of major bleeding^17^ and thus believe that the high quality of INR management provided by CPAS serves as a meaningful and ethical comparator to dabigatran etexilate.

Based upon the power analysis to detect a difference in the primary endpoint, 1100 subjects are required for enrollment for the outcome of dementia. However, this analysis was done for dementia alone and this study will also include serial neurocognitive testing to assess for cognitive decline. For this study, we will enroll 120 subjects. This number of subjects will inform our understanding of the incidence of both dementia and moderate cognitive decline in each anticoagulation group. These data derived from a vanguard analysis, although intentionally underpowered at this time, will help in answering the primary endpoint as well as important other study questions regarding enrollment, retention, and study feasibility.

### 2.3 Study treatments

#### 2.3.1 Dabigatran etexilate dosing

Only currently federal drug association (FDA)‐approved dabigatran etexilate dosing (150 and 75 mg BID) will be administered in this study, with dosing based upon baseline creatinine clearance. Dose adjustments will be made by the Principal Investigator(s) during the study if the patient's renal function changes. For a creatinine clearance of ≥30 mL/min, 150 mg orally BID will be used. For a creatinine clearance of 15 to 30 mL/min, 75 mg orally BID will be used. For a creatinine clearance of <15 mL/min, dabigatran etexilate will be discontinued and anticoagulation will be administered per clinical care. The decision to crossover to warfarin, or to start another anticoagulation agent, will be at the discretion of the Principal Investigator(s) and in agreement with the subject's primary care physician.

#### 2.3.2 Warfarin dosing

Warfarin administration and dosing will be considered standard of care for management of AF with a goal INR of 2‐3. Dose adjustments as well as frequency of anticoagulant effect will be managed through the CPAS.

#### 2.3.3 Post‐study medications

Following the subject's completion of this study, dabigatran etexilate or warfarin will be continued as part of the subjects' standard medical care, as assessed and decided by their cardiologist and/or primary care physician.

### 2.4 Study endpoints and evaluation

**Figure 2:**
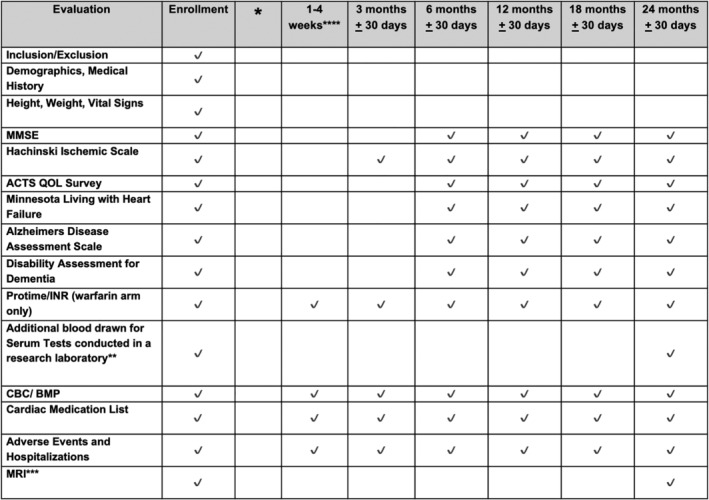
Testing and event assessment schedule for the Cognitive Atrial Fibrillation (CAF) trial including all cognitive testing and laboratory tests performed

Each subject's medical records will be collected as part of usual medical care, prior to enrollment to obtain all baseline demographics relevant up to 6 months prior to study participation. All six questionnaires that are specific to understand cognition, quality of life, and function and how they may interrelate in an anticipated elderly AF population will be administered at the baseline visit, and repeated at the 6‐, 12‐, 18‐, and 24‐month visits as indicated in Figure 2. All testing will be performed by experienced research coordinators that will undergo institutional assessment and certification regarding education, efficacy, and consistency before they begin to administer the tools. These questionnaires are specific to understand cognition, quality of life, function, and how they may interrelate in an anticipated elderly AF population.

The questionnaires and laboratory tests are done to guide subject status, drug dosing, and to assure safety, and will be performed at Intermountain hospitals and/or clinical facilities.

### 2.5 Subgroup analysis

#### 2.5.1 Magnetic resonance imaging (MRI) subgroup

In each group, 10 subjects will be selected to undergo a cranial MRI at baseline to determine brain volume and characteristic changes representative of microbleeding, with a repeat MRI at 24 months. Brain volume will be defined as per routine description.^17^, ^18^, ^19^ More details regarding MRI techniques are contained in Appendix D.

#### 2.5.2 Additional serum tests at baseline

Additional tests that have been linked to the development of stroke, intracranial bleed, and dementia in AF patients, will be performed at enrollment and the 24‐month visit. These tests include the following: brain natriuretic peptide, troponin, growth differentiation factor‐15, cystatin‐C, D‐dimer, matrix metalloprotinease‐9, chemokine ligand 23, endothelial cell adhesion molecule, plasma von Willebrand factor, C reactive protein, prothrombin fragment 1 + 2, P‐selectin, factor VIII, protein C, protein S, anti‐thrombin III, anti‐beta‐2 glycoprotein‐1 IgG and IgM, anticardiolipin IgG and IgM, and lupus anticoagulant panel. Because the most common gene polymorphism associated with sporadic microbleeds is the Apolipoprotein E (APOE) gene on chromosome 19, to control for this variable we will test for this gene as well as and the APOE 2 and 4 alleles which have each been independently associated with lobar microbleeds.^18^, ^19^, ^20^ Neprilysin and the single‐nucleotide polymorphism rs6656401 within the Complement Receptor‐1gene that are associated with severe cerebral amyloid angiopathy will also be assessed.^21^, ^22^

The primary endpoint of the study is the aggregate pf incident dementia or moderate decline in cognitive as determined by serial assessment of the mini mental status examination, the cognitive subscale of the Alzheimer's Disease Assessment Scale (ADAS‐cog11), and the Disability Assessment for Dementia (DAD) test. Further definition of the endpoint criteria, secondary endpoints, and cognitive testing score evaluation is contained in the supplementary file, Appendix C.

### 2.6 Statistical analysis

The study sample size of 120 subjects will permit descriptive statistical analysis to report the feasibility of recruitment, study subject retention, budget adherence and likelihood of obtaining the desired outcomes from the prespecified power analysis, safety, and identification of adverse effects.

General baseline characteristics of the study participants for each of the aims will be described and compared between randomization groups to provide validation of the successful balance of study variables and potential confounders between the groups by the study randomization procedure administered at the time of study enrollment.

For the primary outcomes, 2‐year incident dementia will be evaluated using the chi‐square statistic. Odd ratios will be determined by Logistic regression. The chi‐square statistic will also be utilized to evaluate whether there was a significant change in ADAS‐cog11 (increase of >30%) and DAD (score <50% and/or a 30% decrease) scores. In addition, change scores for ADAS‐cog11 and DAD (2‐year survey score minus baseline survey score) will utilize the student's *t* test. Comparisons of change scores between baseline and other time points (ie, 6, 12, and 18 months) will also use the student's *t* test to determine significant differences at these time points.

Logistic regression will be performed for binary outcomes to calculate odd ratios so that the odds of experiencing an event between arms can be determined. Kaplan‐Meier survival estimate, and log‐rank test will be utilized to determine time to events. Regression analysis will also be utilized to identify clinical and procedural factors that are predictive of the outcomes. Analyses will be performed on an intention‐to‐treat basis.

### 2.7 Study organization

The trial is a single center trial that will be conducted at Intermountain Medical Center in Murray, Utah. The Intermountain Medical Center Cardiovascular Executive Committee and Internal Review Board approved the study. A data safety monitoring board has been created and will review patient events and study progress every 6 months or sooner on an as needed basis. All laboratory samples will be stored and analyzed at Intermountain Medical Center. The CAF trial enrollment is estimated to be complete in the first quarter of 2019.

## 3 RESULTS

The baseline demographics of the study population are shown in Table 1. The first patient was enrolled in March of 2017. As of 27 February 2019, 97 patients have been enrolled. The study populations are very comparable with the exception of higher rates of prior coronary revascularization in the warfarin group. The study is anticipated to end 1 April 2021.

**Table 1:** Baseline demographics of enrolled patients up (as of 27 February 2019)

	Warfarinn = 48	Dabigatrann = 49	*P*‐value
Age (years)	74.5 ± 6.5	74.0 ± 5.5	0.73
Sex (male)	27 (56.3%)	26 (53.1%)	0.75
Prior myocardial infarction	8 (16.7%)	3 (6.1%)	0.15
History of coronary artery disease	16 (33.3%)	8 (16.3%)	0.08
Prior coronary revascularization	6 (12.5%)	1 (2.0%)	0.03
Prior CABG	1 (2.1%)	0 (0%)	0.24
Heart failure	7 (14.6%)	10 (20.4%)	0.47
Prior ablation	2 (4.2%)	5 (10.2%)	0.32
Pacemaker	7 (14.6%)	6 (12.2%)	0.56
Defibrillator	2 (4.2%)	2 (4.1%)	0.81
Diabetes	20 (41.7%)	16 (32.7%)	0.36
Prior stroke	5 (10.4%)	4 (8.2%)	0.61
Prior transient ischemic attack	4 (8.3%)	4 (8.2%)	0.86
History of depression	11 (22.9%)	15 (30.6%)	0.44
History of peripheral vascular disease	5 (10.4%)	3 (6.1%)	0.37
Chronic obstructive pulmonary disease	17 (35.4%)	15 (30.6%)	0.51
Prior cancer	13 (27.1%)	6 (12.2%)	0.06
Type of AF, n = 64			0.74
Paroxysmal	30 (62.5%)	27 (55.1%)	
Persistent	1 (2.1%)	3 (6.1%)	
Long‐standing persistent	1 (2.1%)	2 (4.1%)	
BMI (kg/m^2^), n = 94	29.8 ± 6.9	30.6 ± 9.9	0.66

Abbreviations: AF, atrial fibrillation; BMI, body mass index; CABG, Coronary artery bypass grafting.

## 4 DISCUSSION

A recent consensus statement called for more prospective data on the use, adherence, and efficacy of anticoagulation in AF to prevent cognitive decline and dementia.^23^ As the vast majority of therapies used to halt progression of cognitive decline and dementia have been ineffective, attention has been turned towards identification of high risk patients and prevention.^23^ In the setting of AF patients, prevention strategies under consideration include the role of rhythm control as a means to improve brain perfusion and end organ function as well as early use of anticoagulation. Three trials are designed to specifically examine the anticoagulation type and use as it relates to cognitive function in AF patients. The blinded randomized trial of anticoagulation to prevent ischemic stroke and neurocognitive impairment (BRAIN) AF trial will evaluate the effect of DOAC therapy on cognitive function in patients who otherwise would not warrant oral anticoagulation (ClinicalTrials.gov NCT02387229). The Cognitive Impairment Related to AF Prevention Trial (GIRAF) will determine if DOAC therapy compared to warfarin improves cognitive function at 2 years in patients that traditionally require anticoagulation (ClinicalTrials.gov NCT 01994265). Finally, the CAF trial as discussed herein will specifically evaluate dabigatran etexilate vs warfarin in AF patients as their first line anticoagulant and explore cognitive domains, brain volume and ischemic injury patterns, and biomarkers of injury.

Warfarin use remains the primary method of anticoagulation worldwide and with careful management is associated with low dementia rates. DOAC therapies that favorably impact both embolic and bleeding risk, may provide improved cognitive function in AF patients if the macro events studied in randomized prospective trials signal fewer micro‐bleed and micro‐thrombosis events. Such understanding must come from a prospective randomized trial in which cognition is measured serially, with a battery of tests, and anticoagulation use is carefully described to provide insight into an area of uncertainty.^24^ Although observational data exist, whether DOACs improve long‐term cognitive function in AF is unknown. If proven, early and appropriate use of DOACs will be critical in AF patients to not only reduce risk of stroke, but also dementia and cognitive impairment. These data will continue to have potential impact as wearable technologies alter early diagnosis patterns in patients with and without clinical symptoms.

## CONFLICT OF INTEREST

Thomas Jared Bunch: Research grant (Boehringer Ingelheim, Boston Scientific)—no personal compensation.

John D. Day: Consultant (Boston Scientific, Abbott Medical).

J. Peter Weiss: Consultant (Stereotaxis, Talon Medical).

Scott M. Stevens, Scott C. Woller: Research grant (Pfizer, Bristol Meyers Squib)—no personal compensation.

Victoria Jacobs, Heidi May, Brian Crandall, Michael Cutler, Charles Mallender, Jeff Olson, Jeffrey Osborn: The authors declare no potential conflict of interests.

## Supporting information

Appendices A‐D.
