# Supplementary material for: Rationale and design of the impact of anticoagulation therapy on the Cognitive Decline and Dementia in Patients with Nonvalvular Atrial Fibrillation (CAF) Trial: A Vanguard study
Source: Clin Cardiol. 2019 Apr 10;42(5):506–12. doi: 10.1002/clc.23181 (PMC6522997; doi:10.1002/clc.23181)
Supplement: Supplementary file 1 — Appendices A‐D. [file CLC-42-506-s001.docx]

**Appendix A: Inclusion Criteria**

Eligible patients must meet all of the following criteria

- Male or female >65 years of age.
- Nonvalvular atrial fibrillation (NVAF) documented by electrocardiogram, ambulatory event monitor, or telemetry within 12 months of enrollment. Valvular heart disease as documented by a prior prosthetic heart failure surgery, rheumatic valve disease, of by echocardiogram defining the presence of severity that may prompt need for valve replacement surgery.
- Moderate risk of thromboembolism based upon a CHADS2 score or CHA2DS2-Vasc score of ≥2.
- Ability to complete a mini-mental status evaluation
- Ability to independently comprehend and complete a quality of life and dementia questionnaires.
- Ability to provide informed consent for study participation.
- Willing and able to comply with the prescribed follow-up tests and schedule of evaluations.

**Appendix B. Exclusion Criteria**

Patients will be excluded if they meet any of the following:

1. Inability to take an anticoagulant due to known or perceived bleeding risk.
2. Have a known coagulopathy that may impact the choice, duration, efficacy and safety of anticoagulation therapy.
3. Atrial Fibrillation in the setting of valvular heart disease as defined in the inclusion criteria.
4. Severe renal dysfunction, defined as a creatinine clearance <15 mL/min (documented within the last 3 months).
5. Have a history of any form of dementia formally diagnosed with associated decline in quality of life.
6. Have a life expectancy less than 24 months.
7. Are unable to comply with the follow-up schedule.
8. Are currently participating in a clinical investigation that includes an active pharmacologic treatment arm.
9. An upper age limit not to be used if participation inclusion criteria are met.
10. Participation in any other clinical trials involving investigational or marketed products within 30 days prior to entry in the study.
11. Other conditions that in the opinion of the Principal Investigator(s) may increase risk to the subject and/or compromise the quality of the clinical trial.
12. Concurrent pharmacologic treatment that is required to treat a condition long-term in which concurrent use of dabigatran etexilate is contraindicated.
13. Have received any anticoagulant drug for stroke prevention for more than 90 days.
    - Aspirin and P2Y12 inhibitors (e.g. clopidogrel (Plavix), or prasugrel (Effient)) are not considered anticoagulant drugs.
    - If a patient has received any anticoagulant drug for stroke prevention for less than 90 days, the Principal Investigator(s) or a Co-Investigator will decide the patient’s eligibility for this study

**Appendix C. Endpoint Definition and Assessment**

### Primary Endpoint

1. The primary functional endpoint of this study is to demonstrate that with long-term anticoagulation therapy with dabigatran etexilate (150 mg twice daily (BID) or 75 mg BID, dose based upon renal clearance) will reduce incident dementia and worsening cognitive decline compared to dose-adjusted warfarin.
   1. Evaluating the difference of incident dementia between the treatment arms. Incident dementia will be defined as a formal diagnosis of dementia by a neurologist. In subjects that receive a MMSE score of <24 and report memory impact on quality of life will be referred to neurology for further evaluation of dementia.
   2. Change in cognitive decline. This endpoint will be determined by measuring the change from baseline to study conclusion on the 11-item cognitive subscale of the Alzheimer's Disease Assessment Scale (ADAS-cog11, with scores ranging from 0 to 70 and higher scores indicating greater impairment) and the Disability Assessment for Dementia (DAD, with scores ranging from 0 to 100 and higher scores indicating less impairment). An increase in ADAS-cog11 of >30% is considered significant for moderate cognitive decline. In subjects that score <50% on the DAD, there is a direct correlation with global deterioration scales and scores. Subjects with a 30% decrease in DAD score or those with a score <50% will be considered to have moderate cognitive decline.
2. The primary anatomic endpoint of this study is to demonstrate that with long-term anticoagulation therapy with dabigatran etexilate will reduce micro- and macro-cerebral ischemic events on compared to dose-adjusted warfarin

### Secondary Endpoints

Secondary endpoints of stroke or transient ischemic attack (TIA), intracranial bleed, and changes from baseline scores on the mini-mental status evaluation and the Hachinski Ischemic Scale will be recorded. Secondary outcomes will also include those required to proceed with a potential future larger study such as enrollment potential, feasibility, adherence and dropout rate, and drug tolerability.

The medical records of all patients will be reviewed during the trial to look for adverse events that include any hospitalizations, change in clinical status potentially related to the study therapies, falls or adverse side effects to either therapy outside of bleeding.

**Appendix D. MRI Techniques**

MRI techniques that provide high contrast between brain parenchyma and highly paramagnetic materials (e.g. deoxyhemoglobin, superparamagnetic hemosiderin, and diamagnetic calcium) which are sensitive to rupture of blood vessels as small as 200 µm in diameter are favorable. All MRIs will be performed using the same technology and the same machine as is foreseeably feasible. An MRI protocol that uses T2* Gradient-Recall Echo (T2*GRE) and Susceptibility-Weighted (SWI) MRI techniques to establish baseline imaging evidence of microbleeds and follow-up studies has been shown to achieve these goals.^1-4^ A rating scale will be adopted as described by others that will take into consideration whether lesions are certain or uncertain, and consider them present only if they are observed on serial images. ^5-6^

References

1. Atlas SW, Mark AS, Grossman RI, Gomori JM. Intracranial hemorrhage: gradientecho

MR imaging at 1.5 T. Comparison with spin-echo imaging and clinical applications. *Radiology*.

1988; **168**:803-807.

2. Wu Z, Mittal S, Kish K, Yu Y, Hu J, Haacke EM. Identification of calcification with

MRI using susceptibility-weighted imaging: a case study. *J Magn Reson Imaging*. 2009; **29**:177-182.

3. Tanaka A, Ueno Y, Nakayama Y, Takano K, Takebayashi S. Small chronic

hemorrhages and ischemic lesions in association with spontaneous intracerebral hematomas. *Stroke*.

1999; **30**:1637-1642.

4.Cordonnier C, Potter GM, Jackson CA, et al. Improving interrater agreement about

brain microbleeds: development of the Brain Observer MicroBleed Scale (BOMBS). *Stroke*.

2009; **40**:94-99.

5. Gregoire SM, Chaudhary UJ, Brown MM, et al. The Microbleed Anatomical Rating

Scale (MARS): reliability of a tool to map brain microbleeds. *Neurology*. 2009; **73**:1759-1766.

6.Ayaz M, Boikov AS, Haacke EM, Kido DK, Kirsch WM. Imaging cerebral microbleeds using

susceptibility weighted imaging: one step toward detecting vascular dementia. *J Magn Reson Imaging*.

2010; **31**:142-148.
